# An efficient 3-approximation algorithm for the Steiner tree problem with the minimum number of Steiner points and bounded edge length

**DOI:** 10.1371/journal.pone.0294353

**Published:** 2023-11-21

**Authors:** Donghoon Shin, Sunghee Choi

**Affiliations:** 1 Department of Electrical Engineering and Computer Science, DGIST, Daegu, South Korea; 2 School of Computing, KAIST, Daejeon, South Korea; TU Wien: Technische Universitat Wien, AUSTRIA

## Abstract

We present improved algorithms for the Steiner tree problem with the minimum number of Steiner points and bounded edge length. Given *n* terminal points in a 2D Euclidean plane and an edge length bound, the problem asks to construct a spanning tree of *n* terminal points with minimal Steiner points such that every edge length of the spanning tree is within the given bound. This problem is known to be NP-hard and has practical applications such as relay node placements in wireless networks, wavelength-division multiplexing(WDM) optimal network design, and VLSI design. The best-known deterministic approximation algorithm has *O*(*n*^3^) running time with an approximation ratio of 3. This paper proposes an efficient approximation algorithm using the Voronoi diagram that guarantees an approximation ratio of 3 in *O*(*n* log *n*) time. We also present the first exact algorithm to find an optimal Steiner tree for given three terminal points in constant time. Using this exact algorithm, we improve the 3-approximation algorithm with better performance regarding the number of required Steiner points in *O*(*n* log *n*) time.

## 1 Introduction

In this paper, we present improved algorithms for *the Steiner tree problem with the minimum number of Steiner points and bounded edge length* (STP-MSPBEL) introduced by Lin and Xue [1]. Given a set of *n* terminal points *P* = {*p*_1_, *p*_2_, …, *p*_*n*_} in the two-dimensional Euclidean space $R^{2}$ and a positive constant *R*, STP-MSPBEL asks for a tree spanning a superset of *P* such that each edge in the spanning tree has a length no more than *R* and the number of points other than those in *P*, called *Steiner points* [2], is minimized [1]. This problem has been applied to the relay node placement problem in wireless sensor networks [3, 4]. In monitoring sensor networks, connectivity among sensors is crucial for gathering information. Due to harsh environments like earthquakes, fires, or floods, a number of sensors can be simultaneously disabled. In this scenario, relay sensors are required to restore network connectivity. When the communication distance sets to *R*, STP-MSPBEL can be employed to recover the entire network at a low cost (with a minimal number of relay nodes). This problem also finds applications in wavelength-division multiplexing(WDM) optimal network design, VLSI design, and evolutionary/phylogenetic tree constructions in computational biology [1].

The classical Steiner tree problem, which aims to minimize the total length of the spanning tree, has been widely studied. This problem is NP-hard [5], and it is proven to be APX-hard, so it cannot be approximated within 96/95 in polynomial time unless *p* = *np* [6]. A number of approximation algorithms have been proposed [7–11]. The currently best-known approximation scheme achieved the approximation ratio of ln 4 + *ϵ* ≤ 1.39 using linear programming (LP) and iterative randomized rounding by Byrka et al. [7]. Later, Chen and Hsieh [8] proposed an efficient two-phase heuristic in greedy strategy with an approximation ratio of 1.4295. Recently, Traub and Zenklusen [10] proposed the purely combinatorial approach providing the approximation ratio of ln4+ *ϵ*, which is the same as the best-known approximation factor without solving an LP problem. Zhang et al. [11] presented a truthful-in-expectation mechanism that achieves the same approximation ratio. Berman et al. [12] presented a 1.25-approximation algorithm for the variation of the problem where a complete graph is given with edge lengths 1 and 2. Chen and Hsieh [10] analyzed the algorithm of Byrka et al. [7] and showed that it gives 1.162 + *ϵ* approximation for the problem on complete graphs with edge distances 1 and 2.

In STP-MSPBEL, Lin and Xue [1] proved that the problem is NP-hard and presented a polynomial-time approximation algorithm by exploiting a minimum spanning tree. Let *len*(*e*) be the length of an edge *e*. Given an edge *e*_*i*_ whose length is greater than the given bounded edge length *R*, this algorithm inserts $\lceil \frac{len(e_{i})}{R}\rceil -1$ Steiner points to break the edge *e*_*i*_ into small pieces of length at most *R*. Cheng et al. [3] called such edge as a *steinerized edge*. Given a tree, we can produce a *steinerized tree* for STP-MSPBEL using steinerized edges whenever the edge length exceeds the bounded edge length *R*. Lin and Xue [1] construct a *steinerized minimum spanning tree* from the minimum spanning tree of terminal points [13]. They showed that this algorithm has an approximation ratio of 5. Later, Chen et al. [13] proved that the steinerized minimum spanning tree indeed has an approximation ratio of exactly 4. They also presented a 3-approximation algorithm with *O*(*n*^4^) running time. At initialization, the algorithm adds edges between any pair of two terminal points if the distance between them is less than or equal to *R*. The algorithm examines all possible combinations of four terminal points from the entire set to determine if they are currently not connected and can be connected using a single Steiner point. If so, it inserts one Steiner point and four edges to connect those points and the Steiner point. After examining all subsets of four terminal points, the algorithm connects disconnected components using steinerized edges if the resulting graph is still disconnected. Cheng et al. [3] presented a 3-approximation algorithm with *O*(*n*^3^) running time. Instead of inspecting every subset of four terminal points, they investigate all subsets of three terminal points. Moreover, they proposed a randomized algorithm to give a 2.5 approximation with a probability of at least 0.5 in *poly*(*n*, *q*_*P*_) time where *q*_*P*_ = *max*{*q*_*a*,*b*,*c*_|*a*, *b*, *c* ∈ *P*} and *q*_*a*,*b*,*c*_ is the number of Stiner points used to steinerize the optimum Steiner tree on three terminal points {*a*, *b*, *c*} [3]. Senel and Younis [14] suggested an *O*(*n*^4^)-time algorithm to reduce Steiner points based on the Fermat point for all subsets of three terminal points. Later, they improved the algorithm with the discrete Fermat point in [15]; however, the time complexity of their algorithm to find the discrete Fermat point is not bounded because the algorithm should examine a large number of candidate points when the distance between input points is long. In summary, the best-known approximation ratio of deterministic algorithms for STP-MSPBEL has been 3 with running time *O*(*n*^3^).

We propose an efficient approximation algorithm for STP-MSPBEL using the Voronoi diagram, a well-known geometric data structure in computational geometry. This paper extends our previous work [16]. Our algorithm runs in *O*(*n* log *n*) time with the worst-case approximation ratio of 3, which is the same as the best-known deterministic approximation ratio and remarkably reduces the running time compared to the previous 3-approximation algorithms [3, 13]. In the classical Steiner tree problem, it is known that the optimal Steiner tree for the given three points can be computed in constant time by using the Fermat point [17]; however, in STP-MSPBEL, there has been no exact algorithm even for three terminal points in constant time. After we presented the exact algorithm in our preliminary paper [16], Senel and Younis [18] proposed a simpler method to find the optimal Steiner tree for three terminal points; however, this paper addresses the counterexample that shows their method indeed cannot find the optimal solution. In this paper, we present the exact algorithm to find a Steiner tree for given three points in constant time, and we propose an improved algorithm for STP-MSPBEL by using the exact algorithm. The proposed algorithm ensures a worst-case approximation ratio of 3 in *O*(*n* log *n*) time while achieving improved performance since we utilize the optimal solution of three terminal points.

### Contributions

- We propose an efficient approximation algorithm for STP-MSPBEL using the Voronoi diagram that guarantees an approximation ratio of 3 in *O*(*n* log *n*) time. Previously, the best-known deterministic algorithm has an approximation of 3 in *O*(*n*^3^) time. The proposed algorithm remarkably reduces the running time.
- We present the first exact algorithm to provide an optimal Steiner tree for three input points in constant time. Since no exact algorithm for STP-MSPBEL has been addressed so far, we believe that our algorithm gives a good clue to finding a locally optimal solution. Moreover, it can be used to improve the performance of other approximation algorithms.
- By combining this exact algorithm and the efficient 3-approximation algorithm, we develop another 3-approximation algorithm that runs in *O*(*n* log *n*) time with better performance in terms of the number of required Steiner points.

## 2 Preliminaries

We briefly introduce a Voronoi diagram and the Fermat point, mainly used by the proposed algorithms.

### The Voronoi diagram

Denote the Euclidean distance between two points *p* and *q* by |*pq*|. Let *P* = {*p*_1_, *p*_2_, …, *p*_*n*_} be a set of *n* distinct points in the plane; these points are the sites. A *Voronoi diagram* of *P* denoted by *VD* is defined as a subdivision of the plane into *n* cells, one for each site in *P*, with the property that a point *q* lies in the cell corresponding to a site *p*_*i*_ if and only if |*p*_*i*_*q*| < |*p*_*j*_*q*| for each *p*_*j*_ ∈ *P* with *j* ≠ *i* [19]. In this paper, *VD* indicates only the edges and vertices of the subdivisions. The following properties hold:

**Property 1**. *A point q is a vertex of VD if and only if its latest empty circle of q as its center with respect to P contains three or more sites on its boundary*.

**Property 2**. *The bisector between sites p_i_ and p_j_ defines an edge of VD if and only if there is a point q on the bisector such that its latest empty circle of q as its center with respect to P contains both p_i_ and p_j_ on its boundary but no other site*.

Under the general position assumption that no four sites lie on the same circle, each vertex of the Voronoi diagram has a degree of three which means the Voronoi vertex has equidistance to three relevant sites. The number of Voronoi vertices is at most 2*n* − 5, and the number of Voronoi edges is at most 3*n* − 6. It can be efficiently computed in *O*(*n* log *n*) time [20].

### The Fermat point

The *Fermat point* (or called *Torricelli* point) *p*_*F*_ of a triangle Δ*p*_*a*_*p*_*b*_*p*_*c*_ is a point such that the sum of distances from *p*_*F*_ to each vertex (|*p*_*F*_*p*_*a*_| + |*p*_*F*_*p*_*b*_| + |*p*_*F*_*p*_*c*_|) is minimized. In the classical Steiner tree problem [21], the Fermat point is used to build the optimal Steiner tree for the given three points. There are two cases to compute the Fermat point. In one case where a triangle has an angle $\geq \frac{2}{3}\pi$, the Fermat point is one of the input points; precisely, the Fermat point is sited at the obtuse-angled. In the other case, the Fermat point is inside the triangle Δ*p*_*a*_*p*_*b*_*p*_*c*_ and can be found by drawing equilateral triangles on each side [17]. In this case, the following property holds:

**Property 3**. *The angles subtended by the sides of the triangle at the Fermat point are all equal to* $\frac{2}{3}\pi$ *when the Fermat point is inside the triangle*.

## 3 An efficient 3-approximation algorithm

**Fig 1:**
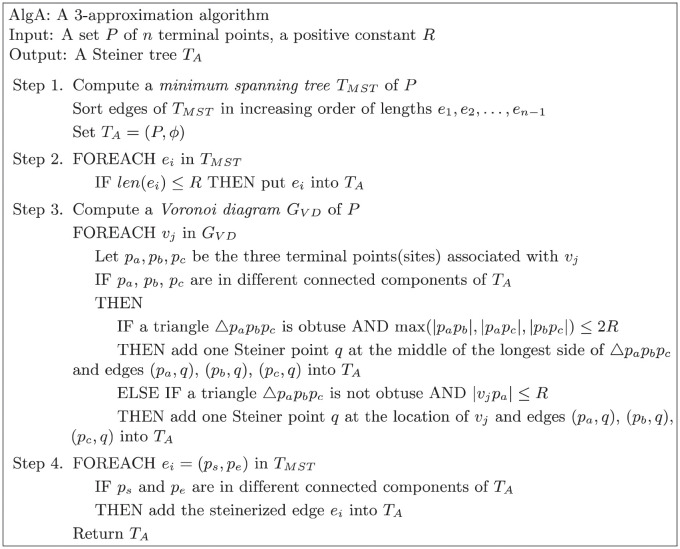
*AlgA*: An efficient 3-approximation algorithm.

We propose an *O*(*n* log *n*)-time approximation algorithm with an approximation ratio of at most three by exploiting the Voronoi diagram as described in Fig 1. More precisely, it takes *O*(*n* log *n*) time to determine the number of Steiner points. It takes *O*(*n* log *n* + *S*) to report the locations of Steiner points where *S* is the number of Steiner points in the optimal Steiner tree for STP-MSPBEL since the algorithm produces no more than three times the number of Steiner points in the optimal Steiner tree. Indeed, the number of Steiner points *S* is independent of the number of terminal points *n*. Similar to the previous works [3, 13], this paper does not consider the cost of making steinerized edges when analyzing algorithms. We first compute a minimum spanning tree and sort the edges in increasing order in Step 1. If the length of the edge is less than or equal to *R*, we insert the edge into the result tree in Step 2. If several connected components still remain after adding edges, it requires Steiner points to connect those components. In Step 3, we construct a Voronoi diagram of the terminal points as sites. At each Voronoi vertex, we investigate its three relevant sites, {*p*_*a*_, *p*_*b*_, *p*_*c*_} ∈ *P*, to determine whether these points are in different connected components and whether the radius of the smallest enclosing circle of three sites is less than *R*. If so, we add one Steiner point at the center of the circle and insert three edges to connect relevant terminal points from the Steiner point. The center of the smallest enclosing circle for *p*_*a*_, *p*_*b*_, and *p*_*c*_ is located at the Voronoi vertex when the triangle Δ*p*_*a*_*p*_*b*_*p*_*c*_ is not an obtuse triangle by property 1. If Δ*p*_*a*_*p*_*b*_*p*_*c*_ is an obtuse triangle, the center of the smallest enclosing circle is located at the middle of the longest side of Δ*p*_*a*_*p*_*b*_*p*_*c*_. Note that if the Voronoi vertex is associated with more than three sites, the same approach can be applied using the same Voronoi vertex. Lastly, if two endpoints of the edges in the minimum spanning tree are in different connected components, we steinerize the edge and insert it into the result tree in Step 4.

Now, we analyze the time complexity and performance of the proposed algorithm.

**Lemma 1**. *AlgA takes O*(*n* log *n*) *time*.

*Proof*. In Step 1, it takes *O*(*n* log *n*) time to compute the minimum spanning tree and to sort the edges of the minimum spanning tree. In Steps 2 and 4, we check at most *n* − 1 edges whether the edge connects two different connected components. Connected components can be efficiently maintained by *disjoint-set forest*, a well-known data structure in which connectivity query can be answered in *O*(log *n*) time, and two trees are merged in *O*(1) time [22]. Consequently, Steps 2 and 4 take *O*(*n* log *n*) time. The Voronoi diagram of *n* sites in the plane can be constructed in *O*(*n* log *n*) time [4]. Since the number of vertices in the Voronoi diagram is at most 2*n* − 5 in 2D, Step 3 also takes in *O*(*n* log *n*) time. Overall, *AlgA* takes *O*(*n* log *n*) time.

We show that the *AlgA* needs Steiner points no more than three times the number of Steiner points in the optimal solution in a similar way of [3, 13]. We briefly introduce the terminology used in the previous work. A *full component* of a Steiner tree is a subtree in which every Steiner point is a non-leaf node, and every input point is a leaf node. Given a Steiner tree *T*, *C*(*T*) denotes the number of Steiner points in *T*.

**Lemma 2**. [1]. *There exists a shortest length optimal Steiner tree for STP-MSPBEL such that every Steiner point has degree at most five*.

**Lemma 3**. [13]. *Every steinerized minimum spanning tree has the minimum number of Steiner points among steinerized spanning trees*.

**Lemma 4**. [13]. Let T* be a shortest optimal tree for STP-MSPBEL with property that every Steiner point has degree at most five. Let T_j_ be a full component of T*. *Then the followings hold*:

(1) *The steinerized minimum spanning tree on terminals in T_j_ contains at most* 3 ⋅ *C*(*T*_*j*_) + 1 *Steiner points*.
(2) *If T_j_ contains a Steiner point with degree at most four, then the steinerized minimum spanning tree on terminals in T_j_ contains at most* 3 ⋅ *C*(*T*_*j*_) *Steiner points*.
(3) *If the steinerized minimum spanning tree on terminals in T_j_ contains an edge between two terminals, then it contains at most* 3 ⋅ *C*(*T*_*j*_) *Steiner points*.

We slightly modify the lemma in [13] to apply it to our algorithm.

**Lemma 5**. Let T* be an optimal tree for STP-MSPBEL *and T_A_ be the tree produced by* AlgA. *Then C*(*T*_*A*_) ≤ 3 ⋅ *C*(T*).

*Proof*. Let *T*_*S*_ be the steinerized minimum spanning tree. Let *k* be the number of Steiner points inserted by *AlgA* in Step 3. Since each of those Steiner points combines three different connected components at once and every edge whose length is less than or equal to *R* in the minimum spanning tree is added in Step 2, it is obvious that

$$\begin{array}{c} C\left(T_{A}\right)\leq C\left(T_{S}\right)-k. \end{array}$$ (1)

By Lemma 2, we assume that T* is the shortest length optimal Steiner tree such that every Steiner point has a degree at most five. We split T* into full components *T*_*j*_(1 ≤ *j* ≤ *N*_*f*_) where *N*_*f*_ is the number of full components. For each full component *T*_*j*_, we construct corresponding steinerized minimum spanning trees $T_{j}^{{}^{\prime }}$. If any full component contains at least one Steiner point whose degree is at most four, the corresponding steinerized minimum spanning tree has not more than 3 times the number of Steiner points in the full components by Lemma 4 (2). Moreover, if the distance between any pair of two points in the full component is not more than *R*, the corresponding steinerized minimum spanning tree requires no more than 3 times the number of Steiner points in the full components by Lemma 4 (3). Now, we consider a spanning tree $T^{\prime }\left(=\cup_{j=1}^{N_{f}}T_{j}^{{}^{\prime }}\right)$ which consists of steinerized minimum spanning trees of each full component. By Lemma 4,

$$\begin{array}{c} C\left(T^{\prime }\right)\leq 3\cdot C\left(T^{*}\right)+g \end{array}$$ (2)

where *g* is the number of full components in which every Steiner point has degree 5 and the distance between any two points is greater than *R*. By Lemma 3,

$$\begin{array}{c} C\left(T_{S}\right)\leq C\left(T^{\prime }\right). \end{array}$$ (3)

From (1), (2) and (3), *C*(*T*_*A*_) ≤ 3 ⋅ *C*(T*) + *g* − *k*. Therefore, the approximation ratio produced by *AlgA* is bounded by 3 if *g* ≤ *k*. To show that *g* ≤ *k*, we construct two forests *F*_1_ and *F*_2_. We put all input points into *F*_1_ and *F*_2_ each and then add edges whose length is less than or equal to *R* in the minimum spanning tree. Let *p* be the number of connected components in *F*_1_ (= *F*_2_) so far, which is the result of Step 2 of *AlgA*. We add *k* Steiner points and their induced edges to *F*_1_ according to Step 3. The number of connected components in *F*_1_ is equal to *p* − 2*k*.

Now, we examine *g* full components in which every Steiner point has a degree of 5. Such components are either only one Steiner point or multiple Steiner points. In the case of components with a single Steiner point in which 5 input points are in different connected components and enclosed by a circle with a radius of *R*, the input points belong to at most three connected components in *F*_1_ after Step 3. We deal with three Voronoi vertices related 5 input points in Step 3. Let us consider one of Voronoi vertices and its relevant three points. If they are in all the different components, they are connected by a Steiner point. Otherwise, at least two of the points are already connected. Then we look into another Voronoi vertex associated with two input points unrelated to the former Voronoi vertex. Similarly, at least two of these points become a part of the same component. Accordingly, we connect two pairs of input points by adding two edges in *F*_2_. Even though we merge them, the number of connected components in *F*_2_ is still greater than or equal to that in *F*_1_. Now, we consider the other case. If the full component has more than one Steiner point, we can always find two Steiner points whose four neighbor points are input points. These four points belong to at most three connected components in *F*_1_ after Step 3. In a similar way to the previous case, at least three points are examined for connectivity due to the associated Voronoi vertex in Step 3. A Steiner point connects them, or two of them are already connected. Even if we add two edges into *F*_2_ to connect a pair of input points at each of two Steiner points, the number of connected components in *F*_2_ is still greater than or equal to that in *F*_1_. In both cases, the number of connected components is equal to *p* − 2*g* in *F*_2_ since we add two edges for every *g* full component. It satisfies that *p* − 2*k* ≤ *p* − 2*g*. Therefore, *g* ≤ *k*.

Lemmas 1 and 5 yield the following theorem.

**Theorem 1**. AlgA *produces a Steiner tree in which the number of Steiner points is no more than 3 times the number of Steiner points in the optimal Steiner tree in O*(*n* log *n*) *time*.

## 4 An exact algorithm for three input points

**Fig 2:**
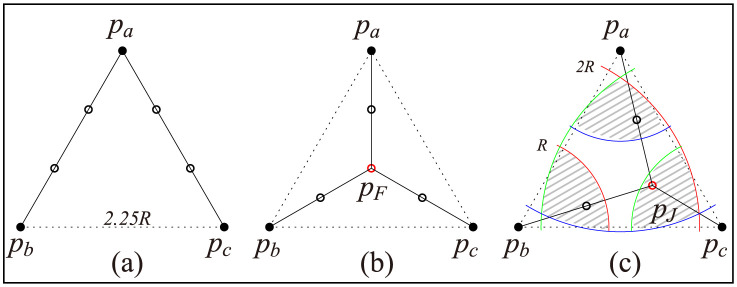
Black dots represent input terminal points *p*_*a*_, *p*_*b*_, and *p*_*c*_. Empty dots represent Steiner points. (a) the steinerized minimum spanning tree requires 4 Steiner points (b) the steinerized 3-star tree using the Fermat point requires 4 Steiner points (c) the optimal Steiner tree for STP-MSPBEL requires 3 Steiner points. A junction point can be optimally placed in any of 3 shaded regions.

Given three terminal points *p*_*a*_, *p*_*b*_, and *p*_*c*_, we present the first exact algorithm *AlgB* to determine the optimal number of Steiner points for STP-MSPBEL in constant time as well as we provide one of the optimal Stiner trees. An optimal tree connecting *p*_*a*_, *p*_*b*_, and *p*_*c*_ is formed as either a 3-star or a wedge, as shown in Fig 2. If an optimal Steiner tree is shaped as a wedge, we can easily find the optimal tree because the steinerized minimum spanning tree becomes optimal by Lemma 3. In the other case, *p*_*a*_, *p*_*b*_, and *p*_*c*_ are connected by a *junction point* *p*_*J*_, which is a tree in the shape of a 3-star. In this case, it is important to seek the location of *p*_*J*_ at which $\left\lceil \frac{|p_{a}p_{J}|}{R}\right\rceil +\left\lceil \frac{|p_{b}p_{J}|}{R}\right\rceil +\left\lceil \frac{|p_{c}p_{J}|}{R}\right\rceil$ is minimized. Since the optimal Steiner tree is formed as either a wedge, a 3-star, or both, it can be obtained by comparing the 3-star and the wedge. Since finding a steinerized minimum spanning tree is trivial for three points, the main part of *AlgB* aims to seek an optimal junction point.

The Fermat point *p*_*F*_ can be a good candidate as a junction point of *p*_*a*_, *p*_*b*_, and *p*_*c*_ because the Fermat point is defined as a point that minimizes |*p*_*a*_*p*_*F*_| + |*p*_*b*_*p*_*F*_| + |*p*_*c*_*p*_*F*_|. In STP-MSPBEL, unfortunately, *p*_*F*_ does not always become an optimal location for the junction point, as shown in Fig 2. Consider an equilateral triangle formed by *p*_*a*_, *p*_*b*_, and *p*_*c*_ where each length of a side is 2.25*R*. The steinerized minimum spanning tree and a steinerized 3-star tree using the Fermat point require four Steiner points to satisfy bounded edge length *R*. The optimal Steiner tree, however, needs three Steiner points, as shown in Fig 2(c).

Let *T*_*F*_ be a 3-star tree connecting input points *p*_*a*_, *p*_*b*_, and *p*_*c*_ using the Fermat point *p*_*F*_ as a junction point *p*_*J*_.

**Lemma 6**. *There is no steinerized 3-star tree T*_3*s*_ *such that C*(*T*_3*s*_) < *C*(*T*_*F*_) − 2.

*Prrof*. It is obvious that $\left\lceil \frac{|p_{a}p_{F}|}{R}\right\rceil +\left\lceil \frac{|p_{b}p_{F}|}{R}\right\rceil +\left\lceil \frac{|p_{c}p_{F}|}{R}\right\rceil -3<\frac{|p_{a}p_{F}\left|+\right|p_{b}p_{F}\left|+\right|p_{c}p_{F}|}{R}\leq \left\lceil \frac{|p_{a}p_{F}|}{R}\right\rceil +\left\lceil \frac{|p_{b}p_{F}|}{R}\right\rceil +\left\lceil \frac{|p_{c}p_{F}|}{R}\right\rceil$ and $C\left(T_{F}\right)=\left\lceil \frac{|p_{a}p_{F}|}{R}\right\rceil +\left\lceil \frac{|p_{b}p_{F}|}{R}\right\rceil +\left\lceil \frac{|p_{c}p_{F}|}{R}\right\rceil -2$. Suppose that there is a steinerized 3-star tree *T*_3*s*_ such that *C*(*T*_3*s*_) < *C*(*T*_*F*_) − 2. Let *p*_*J*′_ be a junction point of *T*_3*s*_. To satisfy *C*(*T*_3*s*_) < *C*(*T*_*F*_) − 2, the following inequalities should hold: $\frac{|p_{a}p_{J^{\prime }}\left|+\right|p_{b}p_{J^{\prime }}\left|+\right|p_{c}p_{J^{\prime }}|}{R}\leq \left\lceil \frac{|p_{a}p_{J^{\prime }}|}{R}\right\rceil +\left\lceil \frac{|p_{b}p_{J^{\prime }}|}{R}\right\rceil +\left\lceil \frac{|p_{c}p_{J^{\prime }}|}{R}\right\rceil \leq \left\lceil \frac{|p_{a}p_{F}|}{R}\right\rceil +\left\lceil \frac{|p_{b}p_{F}|}{R}\right\rceil +\left\lceil \frac{|p_{c}p_{F}|}{R}\right\rceil -3$. Since *T*_*F*_ minimizes the sum of edge lengths, $\left\lceil \frac{|p_{a}p_{F}|}{R}\right\rceil +\left\lceil \frac{|p_{b}p_{F}|}{R}\right\rceil +\left\lceil \frac{|p_{c}p_{F}|}{R}\right\rceil -3<\frac{|p_{a}p_{F}\left|+\right|p_{b}p_{F}\left|+\right|p_{c}p_{F}|}{R}<\frac{|p_{a}p_{J^{\prime }}\left|+\right|p_{b}p_{J^{\prime }}\left|+\right|p_{c}p_{J^{\prime }}|}{R}$. It contradicts that $\left\lceil \frac{|p_{a}p_{J^{\prime }}|}{R}\right\rceil +\left\lceil \frac{|p_{b}p_{J^{\prime }}|}{R}\right\rceil +\left\lceil \frac{|p_{c}p_{J^{\prime }}|}{R}\right\rceil \leq \left\lceil \frac{|p_{a}p_{F}|}{R}\right\rceil +\left\lceil \frac{|p_{b}p_{F}|}{R}\right\rceil +\left\lceil \frac{|p_{c}p_{F}|}{R}\right\rceil -3$. Thus, such *T*_3*s*_ cannot exist.

Let $C_{x}^{y}$ be a circle centered at a point *p*_*x*_ with a radius of *y* ⋅ *R*. We subdivide the plane by circles centered at each input point *p*_*a*_, *p*_*b*_, and *p*_*c*_ with various radii; we draw concentric circles $C_{a}^{q}$, $C_{b}^{q}$, and $C_{c}^{q}$, q∈N as illustrated in Fig 4.

**Observation 1**. *When the optimal Steiner tree of three points is formed as 3-star, the optimal location of p_J_ is inside the convex cell, which is an intersection area of* $C_{a}^{i^{*}}$, $C_{b}^{j^{*}}$, *and* $C_{c}^{k^{*}}$ such that i* + j* + k* *is minimized. Note that an optimal convex cell can be a single point if circles intersect at a point*.

*Proof*. It is obvious that any point *p*_*x*_ in $C_{a}^{q}$ (or $C_{b}^{q}$, $C_{c}^{q}$) requires at least *q* − 1 Steiner points to reach *p*_*a*_ (or *p*_*b*_, *p*_*c*_). When *p*_*x*_ is a junction point and $C_{a}^{i}$, $C_{b}^{j}$, and $C_{c}^{k}$ are the smallest circles containing *p*_*x*_, we need *i* + *j* + *k* − 2 Steiner points including *p*_*x*_ to connect all input points. If *p*_*x*_ is not in the convex cell, we can always find the cell in which any point *p*_*x*′_ can connect *p*_*a*_, *p*_*b*_, and *p*_*c*_ with fewer Steiner points. Suppose that the cell containing *p*_*x*_ has at least one concave arc on its boundary and w.l.o.g., we assume that this arc is the part of circle $C_{a}^{i-1}$. Since *p*_*x*_ is outside of $C_{a}^{i-1}$, any point *p*_*x*′_ contained by $C_{a}^{i-1}$, $C_{b}^{j}$, and $C_{c}^{k}$ requires one fewer Steiner points than that of *p*_*x*_. Therefore, an optimal junction point *p*_*J*_ should be inside of the convex region generated by $C_{a}^{i^{*}}$, $C_{b}^{j^{*}}$, and $C_{c}^{k^{*}}$ such that i* + j* + k* is minimum.

Based on Observation 1, it is easy to show that the optimal location of a junction point can be represented as a region rather than a single point. Therefore, we focus on searching for a convex region induced by the intersection of circles; more precisely, we search for an optimal region among convex cells in the plane subdivided by concentric circles.

**Observation 2**. *An optimal convex cell may not be unique*.

In the example of Fig 2(c), there are three cells in which we can optimally put a junction point. As the distance between terminal points increases, the number of convex regions requiring the same number of Steiner points can be highly many. Indeed, the number of optimal regions is not bounded by the input size. In this paper, we provide an algorithm to find at least one optimal convex cell to construct the Steiner tree, which requires the least number of Steiner points among all possible 3-star trees connecting *p*_*a*_, *p*_*b*_, and *p*_*c*_.

**Observation 3**. *The intersection of triangle* Δ*p*_*a*_*p*_*b*_*p*_*c*_ *and optimal regions for the junction point of p*_*a*_, *p*_*b*_, *and p*_*c*_ *is not empty*.

*Proof*. Let $\bar{pq}$ be a line through two points *p* and *q*. Suppose that an optimal convex cell is completely outside of Δ*p*_*a*_*p*_*b*_*p*_*c*_ and w.l.o.g., this convex cell lies on the opposite side of $\bar{p_{a}p_{b}}$ from *p*_*c*_. The boundary of the convex cell does not consist of $C_{c}^{k},k\in N$ since *p*_*c*_ is on the opposite side. If the convex cell consists of $C_{a}^{i}$ and $C_{b}^{j}$, it should stab the line $\bar{p_{a}p_{b}}$. Therefore, the optimal convex cells are either completely inside of Δ*p*_*a*_*p*_*b*_*p*_*c*_ or on the edges of Δ*p*_*a*_*p*_*b*_*p*_*c*_.

**Fig 3:**
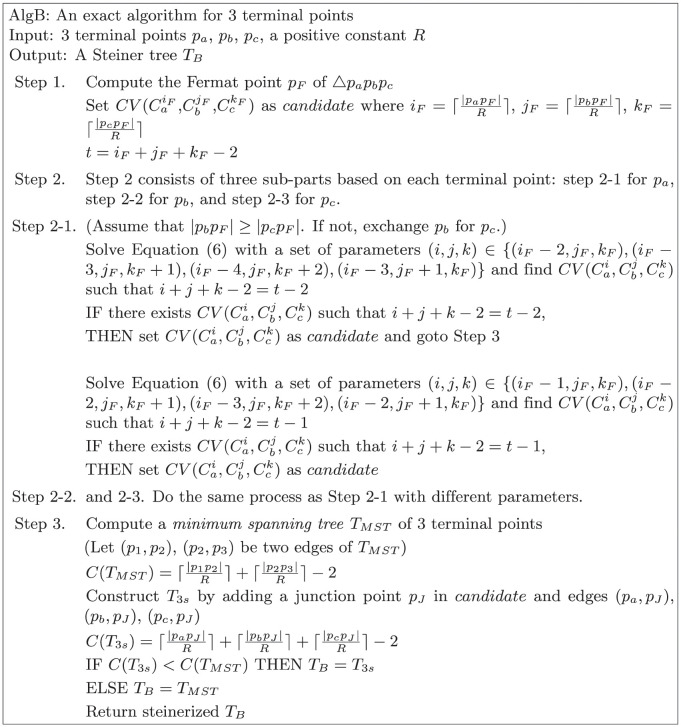
*AlgB*: An exact algorithm for three terminal points.

We present *AlgB*, which provides an optimal Steiner tree for three input points as described in Fig 3. We consider a 3-star tree first and then compare it with the minimum spanning tree. The convex region formed by the intersection of three circles $C_{a}^{i}$, $C_{b}^{j}$, and $C_{c}^{k}$ is denoted by $CV(C_{a}^{i},C_{b}^{j},C_{c}^{k})$. Let $I_{a}\left(C_{b}^{j},C_{c}^{k}\right)$ be the intersection point of $C_{b}^{j}$ and $C_{c}^{k}$ that lies on the same side of $\bar{p_{b}p_{c}}$ as *p*_*a*_ or on $\bar{p_{b}p_{c}}$, in other words, the intersection point closer to *p*_*a*_.

In the first phase, we aim to construct an optimal 3-star tree which induces a Steiner tree requiring the minimum number of Steiner points under the bounded edge length *R* among all possible 3-star trees (refer to Step 1 and 2 of *AlgB* in Fig 3). By Observations 1 and 3, we can find the optimal location for the junction point by checking all convex cells in Δ*p*_*a*_*p*_*b*_*p*_*c*_. Because there could be a number of optimal regions in Δ*p*_*a*_*p*_*b*_*p*_*c*_ by Observation 2, the algorithm focuses on finding one optimal convex cell. The first phase consists of three sub-parts symmetrically in which each input point is used as a reference point. W.l.o.g., we explain the sub-part on the basis of a point *p*_*a*_. By Lemma 6, the number of Steiner points for an optimal 3-star tree is between *i*_*F*_ + *j*_*F*_ + *k*_*F*_ − 4 and *i*_*F*_ + *j*_*F*_ + *k*_*F*_ − 2 where $i_{F}=\left\lceil \frac{|p_{a}p_{F}|}{R}\right\rceil$, $j_{F}=\left\lceil \frac{|p_{b}p_{F}|}{R}\right\rceil$, and $k_{F}=\left\lceil \frac{|p_{c}p_{F}|}{R}\right\rceil$. In the case that the optimal number of Steiner points is equal to *i*_*F*_ + *j*_*F*_ + *k*_*F*_ − 2, the Fermat point becomes one of the optimal junction points. Otherwise, the algorithm searches for the convex cell $CV(C_{a}^{i},C_{b}^{j},C_{c}^{k})$ such that *i*+ *j*+ *k*−2 is equal to *i*_*F*_+ *j*_*F*_+ *k*_*F*_−3 or *i*_*F*_+ *j*_*F*_+ *k*_*F*_−4. We first assume that the optimal 3-star tree requires *i*_*F*_ + *j*_*F*_ + *k*_*F*_ − 4 Steiner points. To figure out the existence of the target convex cell, we consider the series of particular convex cells which form $CV(C_{a}^{i-2\delta_{z}},C_{b}^{j+\delta_{z}},C_{c}^{k+\delta_{z}})$ where *i* + *j* + *k* = *i*_*F*_ + *j*_*F*_ + *k*_*F*_ − 2 and $i,j,k,\delta_{z}\in Z$. When *j* and *k* are given, we check whether there exists *δ*_*z*_ satisfying that $CV(C_{a}^{i-2\delta_{z}},C_{b}^{j+\delta_{z}},C_{c}^{k+\delta_{z}})$ is not empty where *i* = *i*_*F*_ + *j*_*F*_ + *k*_*F*_ − *j* − *k* − 2. We build a distance function $d\left(j,k,\delta_{r}\right),\delta_{r}\in R$ that signifies the distance between *p*_*a*_ and $I_{a}\left(C_{b}^{j+\delta_{r}},C_{c}^{k+\delta_{r}}\right)$. If there exists any integer value $\delta_{z}^{*}$ satisfying $d\left(j,k,\delta_{z}^{*}\right)\leq \left(i-2\delta_{z}^{*}\right)R$, there exists a convex cell $CV(C_{a}^{i-2\delta_{z}^{*}},C_{b}^{j+\delta_{z}^{*}},C_{c}^{k+\delta_{z}^{*}})$. Given *i*, *j*, and *k*, we can find the interval of *δ*_*r*_ to satisfy the following inequality:

$$\begin{array}{c} f\left(i,j,k,\delta_{r}\right)=d\left(j,k,\delta_{r}\right)-\left(i-2\delta_{r}\right)R\leq 0. \end{array}$$ (4)

If the intervals of *δ*_*r*_ satisfying *f*(*i*, *j*, *k*, *δ*_*r*_) ≤ 0 include any integer value $\delta_{z}^{*}$, $CV(C_{a}^{i-2\delta_{z}^{*}},C_{b}^{j+\delta_{z}^{*}},C_{c}^{k+\delta_{z}^{*}})$ is nominated for a candidate cell. If there is no convex cell satisfying *i* + *j* + *k* − 2 = *i*_*F*_ + *j*_*F*_ + *k*_*F*_ − 4, the algorithm seeks convex cells which require *i*_*F*_ + *j*_*F*_ + *k*_*F*_ − 3 Steiner points in the same manner.

For easy representation, we use the similarity transform by setting *p*_*b*_ = (0, 0) and *p*_*c*_ = (1, 0). The scale factor of the transform is $\frac{1}{|p_{b}p_{c}|}$. Accordingly, *p*_*a*_ is transformed to $p_{a}^{\prime }=\left(x_{a},y_{a}\right)$ and bounded edge length *R* becomes $R^{\prime }=\frac{R}{|p_{b}p_{c}|}$. For given *j* and *k*, the coordinate of transformed $I_{a}\left(C_{b}^{j+\delta_{r}},C_{c}^{k+\delta_{r}}\right)$ is represented by (*x*_*i*_, *y*_*i*_) where $x_{i}=\frac{{\left(jR^{\prime }+\delta_{r}R^{\prime }\right)}^{2}-{\left(kR^{\prime }+\delta_{r}R^{\prime }\right)}^{2}+1}{2}$ and $y_{i}=\sqrt{{\left(jR^{\prime }+\delta_{r}R^{\prime }\right)}^{2}-x_{i}^{2}}$. Now, we rewrite the function *f*(*i*, *j*, *k*, *δ*_*r*_) in (4) as follows.

$$\begin{array}{c} f^{\prime }\left(i,j,k,\delta_{r}\right)=d^{\prime }\left(j,k,\delta_{r}\right)-\left(i-2\delta_{r}\right)R^{\prime } \end{array}$$ (5)

where $d^{\prime }\left(j,k,\delta_{r}\right)=\sqrt{{\left(x_{i}-x_{a}\right)}^{2}+{\left(y_{i}-y_{a}\right)}^{2}}$.

By substituting $\delta_{r}^{\prime }-\frac{j+k}{2}+\frac{1}{2R^{\prime }}$ for *δ*_*r*_, we can rewrite the function *f*′(*i*, *j*, *k*, *δ*_*r*_) as follows:

$$\begin{array}{c} f^{\prime }\left(i,j,k,\delta_{r}^{\prime }\right)=\sqrt{{\left(\left(\left(2a-1\right)R^{\prime }\delta_{r}^{\prime }+a\right)-x_{a}\right)}^{2}+{\left(2\sqrt{a\left(1-a\right)R^{\prime }\delta_{r}^{\prime }\left(1+R^{\prime }\delta_{r}^{\prime }\right)}-y_{a}\right)}^{2}}\\ \;-R^{\prime }\left(t+2-2\delta_{r}^{\prime }\right)+1 \end{array}$$ (6)

where $a=\frac{\left(j-k\right)R^{\prime }+1}{2}$ and *t* is the desired number of Steiner points. Note that *t* is initially set to *i*_*F*_ + *j*_*F*_ + *k*_*F*_ − 4 and later is set to *i*_*F*_ + *j*_*F*_ + *k*_*F*_ − 3. We can solve Eq (6) by the quartic formula since $f^{\prime }\left(i,j,k,\delta_{r}^{\prime }\right)$ is of quartic form of $\delta_{r}^{\prime }$ for given *i*, *j*, and *k*. We calculate the valid interval of *δ*_*r*_ from $\delta_{r}^{\prime }$ satisfying inequality (7). If we find an integer value $\delta_{z}^{*}$ in the valid interval of *δ*_*r*_, $CV(C_{a}^{i-2\delta_{z}^{*}},C_{b}^{j+\delta_{z}^{*}},C_{c}^{k+\delta_{z}^{*}})$ is set to a candidate cell.

$$\begin{array}{c} f^{\prime }\left(i,j,k,\delta_{r}^{\prime }\right)\leq 0. \end{array}$$ (7)

Once we set the desired number of Steiner points *t*, we should investigate $CV(C_{a}^{i-2\delta_{z}},C_{b}^{j+\delta_{z}},C_{c}^{k+\delta_{z}})$ with all possible combinations of *j* and *k*. In this paper, we reveal that only four pairs of *j* and *k* are enough to find the optimal cell for each of the two *i* values in each sub-part. In other words, instead of checking all convex cells, we solve Eq (6) at most 8 times in each sub-part with the parameters below. W.l.o.g., we assume that |*p*_*b*_*p*_*F*_| ≥ |*p*_*c*_*p*_*F*_|. If |*p*_*b*_*p*_*F*_| < |*p*_*c*_*p*_*F*_|, we regard *p*_*b*_ as *p*_*c*_ and *p*_*c*_ as *p*_*b*_. In the case that the target number of Steiner points *t* is *i*_*F*_ + *j*_*F*_ + *k*_*F*_ − 4, we assign a set of parameters for Eq (6) to (*i*, *j*, *k*) ∈ {(*i*_*F*_ − 2, *j*_*F*_, *k*_*F*_), (*i*_*F*_ − 3, *j*_*F*_, *k*_*F*_ + 1), (*i*_*F*_ − 4, *j*_*F*_, *k*_*F*_ + 2), (*i*_*F*_ − 3, *j*_*F*_ + 1, *k*_*F*_)}. If there exists any convex cell satisfying the condition, the convex cell becomes the optimal solution for the 3-star tree. Otherwise, we increase the *i* value of the above set of parameters by 1 and solve the equations again in order to examine whether there exists a 3-star tree requiring *i*_*F*_ + *j*_*F*_ + *k*_*F*_ − 3 Steiner points. If there is still no convex cell satisfying the condition, $CV(C_{a}^{i_{F}},C_{b}^{j_{F}},C_{c}^{k_{F}})$ becomes the optimal solution for the 3-star tree.

In the second phase, we construct the minimum spanning tree *T*_*MST*_ of terminal points as described in Step 3. If the number of required Steiner points of *T*_*MST*_ is less than that of the optimal 3-star tree *T*_3*S*_, we return steinerized *T*_*MST*_ as an optimal Steiner tree for the three terminal points. Otherwise, we return steinerized *T*_3*S*_.

**Fig 4:**
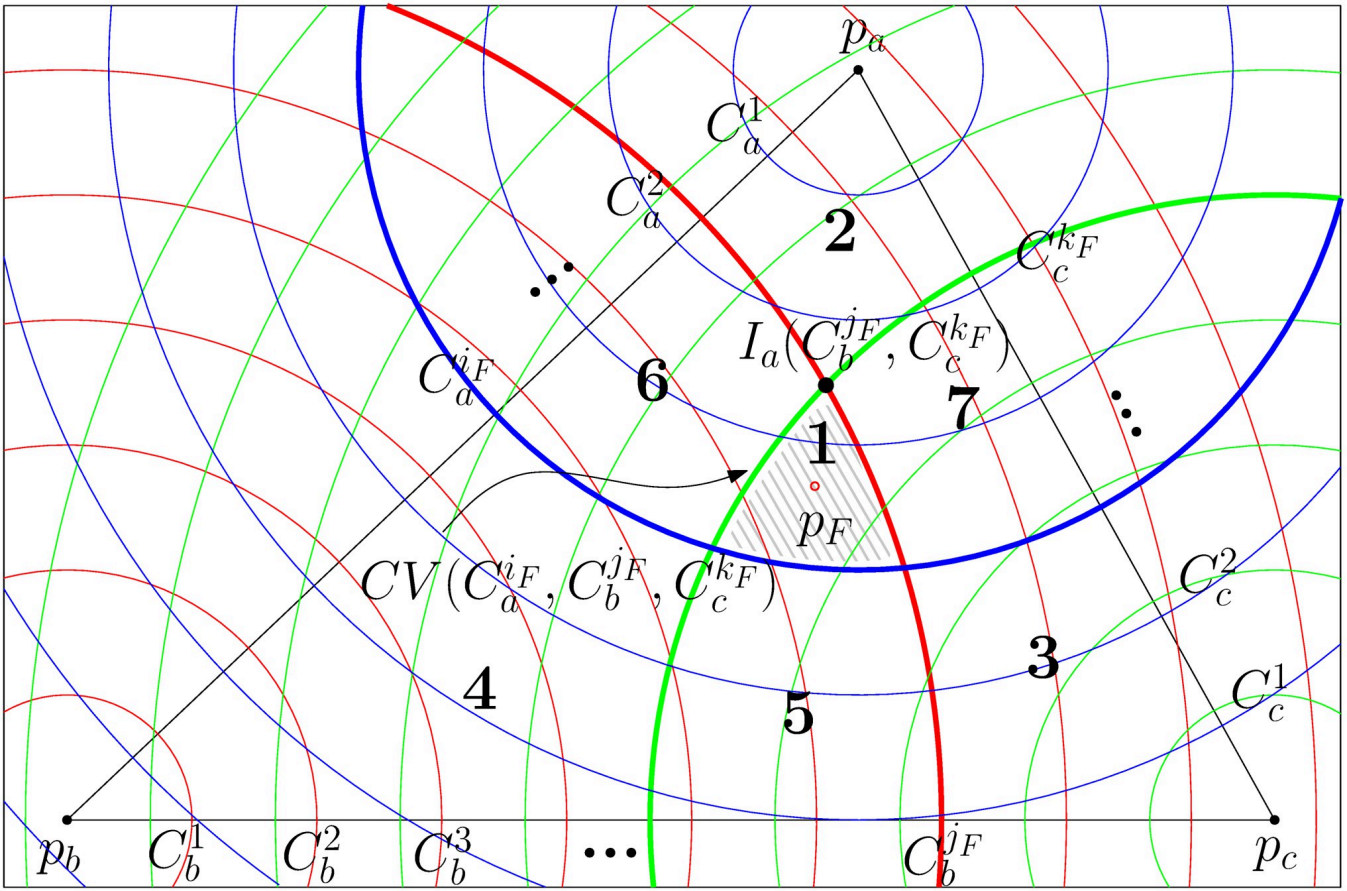
The optimal location for a junction point of a 3-star tree can be identified within the convex cells formed by concentric circles centered at each terminal point. The triangle Δ*p*_*a*_*p*_*b*_*p*_*c*_ is partitioned into 7 regions by $C_{a}^{i_{F}}$, $C_{b}^{j_{F}}$, and $C_{c}^{k_{F}}$.

Now, we prove that the result tree of *AlgB* is an optimal Steiner tree for STP-MSPBEL given three terminal points. We begin by demonstrating that the steinerized 3-star tree, as generated through Step 2, demands the fewest Steiner points compared to all possible 3-star trees connecting terminal points. This is achieved by comparing the candidate convex cell produced by *AlgB* with all convex cells that are in the triangle Δ*p*_*a*_*p*_*b*_*p*_*c*_ or intersect with the boundary of the triangle Δ*p*_*a*_*p*_*b*_*p*_*c*_. We partition the triangle Δ*p*_*a*_*p*_*b*_*p*_*c*_ into seven distinct regions, delineated by $C_{a}^{i_{F}}$, $C_{b}^{j_{F}}$, and $C_{c}^{k_{F}}$ as illustrated in Fig 4. Subsequently, we investigate convex cells within each respective region.

First, we look into region 1, the intersection area of $C_{a}^{i_{F}}$, $C_{b}^{j_{F}}$, and $C_{c}^{k_{F}}$, which indicates the smallest convex cell containing the Fermat point.

**Lemma 7**. *No convex cell* $CV(C_{a}^{i},C_{b}^{j},C_{c}^{k})$ *exists such that i* + *j* + *k* < i* + j* + k*, *i* ≤ *i*_*F*_, *j* ≤ *j*_*F*_, *and k* ≤ *k*_*F*_ *where* $CV(C_{a}^{i^{*}},C_{b}^{j^{*}},C_{c}^{k^{*}})$ *is the candidate cell for the junction point of a 3-star tree as the result of* AlgB.

*Proof*. In region 1, a convex cell $CV(C_{a}^{i},C_{b}^{j},C_{c}^{k})$ satisfies that *i* + *j* + *k* ≥ *i*_*F*_ + *j*_*F*_ + *k*_*F*_ − 2, *i*_*F*_ − 2 ≤ *i* ≤ *i*_*F*_, *j*_*F*_−2 ≤ *j* ≤ *j*_*F*_, and *k*_*F*_ − 2 ≤ *k* ≤ *k*_*F*_ by Lemma 6. In Step 2-1 of *AlgB*, convex cells $CV\left(C_{a}^{i_{F}-\delta 1},C_{b}^{j_{F}-\delta 2},C_{c}^{k_{F}-\delta 2}\right),\delta 1\in \left\{0,1,2\right\},\delta 2\in \left\{0,1,2\right\}$ are investigated by Inequality (7) with parameters (*i*, *j*, *k*) ∈ {(*i*_*F*_ − 2, *j*_*F*_, *k*_*F*_), (*i*_*F*_ − 1, *j*_*F*_, *k*_*F*_)}. Also, convex cells $CV(C_{a}^{i_{F}-\delta 2},C_{b}^{j_{F}-\delta 1},C_{c}^{k_{F}-\delta 2})$ and $CV\left(C_{a}^{i_{F}-\delta 2},C_{b}^{j_{F}-\delta 2},C_{c}^{k_{F}-\delta 1}\right),\delta 1\in \left\{0,1,2\right\},\delta 2\in \left\{0,1,2\right\}$ are checked in Step 2-2 and 2-3 respectively. Consequently, all convex cells are investigated in $CV(C_{a}^{i_{F}},C_{b}^{j_{F}},C_{c}^{k_{F}})$ by *AlgB*.

**Fig 5:**
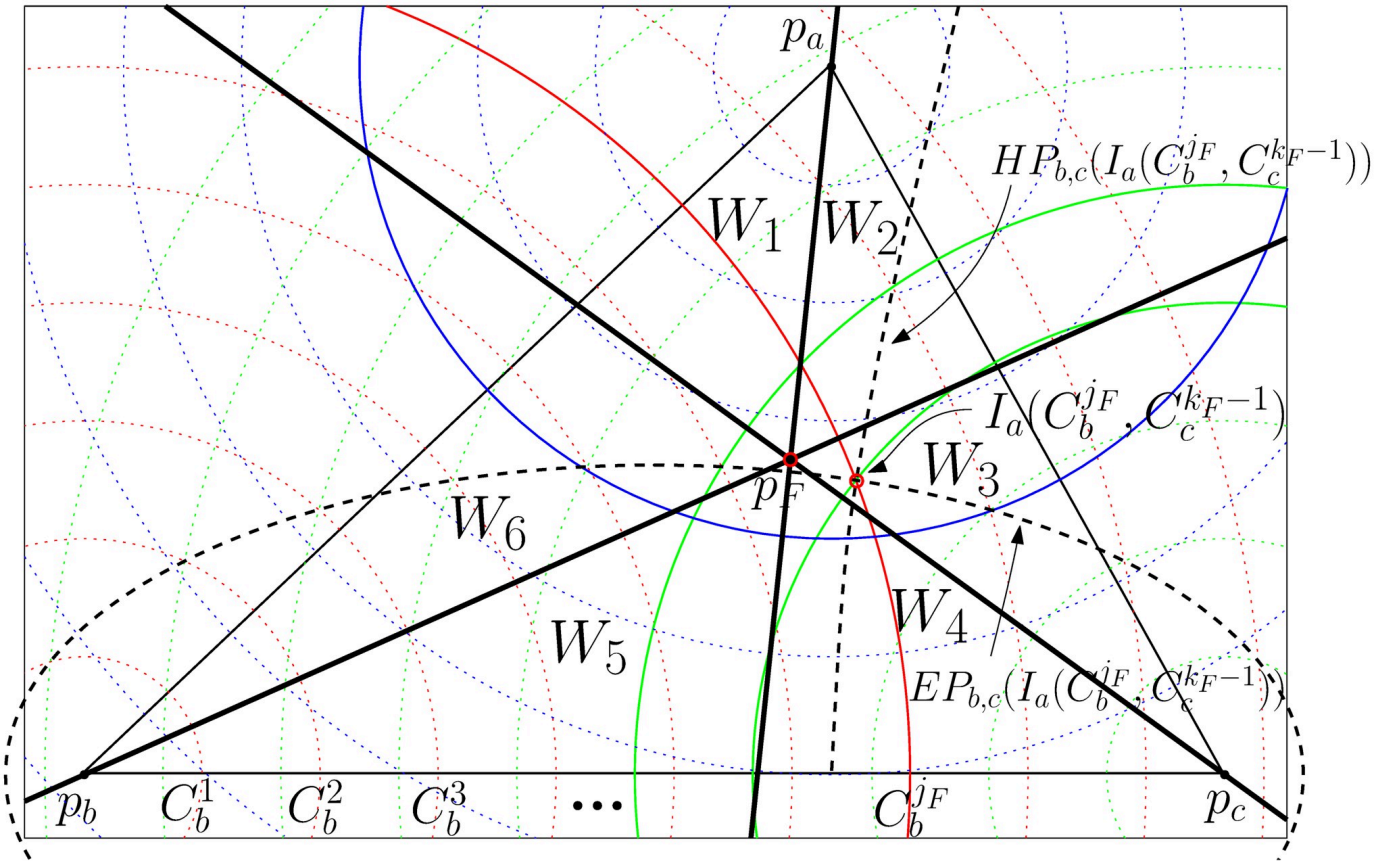
6 wedges (*W*_1_, ⋯, *W*_6_) are delineated by lines $\bar{p_{a}p_{F}}$, $\bar{p_{b}p_{F}}$, and $\bar{p_{c}p_{F}}$ (thick lines). $I_{a}\left(C_{b}^{j_{F}},C_{c}^{k_{F}-1}\right)$ denotes an intersection point of circles $C_{b}^{j_{F}}$ and $C_{c}^{k_{F}-1}$, positioned closer to *p*_*a*_. Dotted curves represent an ellipse $EP_{b,c}\left(I_{a}\left(C_{b}^{j_{F}},C_{c}^{k_{F}-1}\right)\right)$ and a hyperbola $HB_{b,c}\left(I_{a}\left(C_{b}^{j_{F}},C_{c}^{k_{F}-1}\right)\right)$ with two focal points *p*_*b*_ and *p*_*c*_ through a point $I_{a}\left(C_{b}^{j_{F}},C_{c}^{k_{F}-1}\right)$.

To prove Lemmas 8 and 9, we partition the plane into six wedges centered around the Fermat point *p*_*F*_ using the lines $\bar{p_{a}p_{F}}$, $\bar{p_{b}p_{F}}$, and $\bar{p_{c}p_{F}}$. Note that each wedge has an interior angle of 60°. As illustrated in Fig 5, we denote *W*_1_ as the wedge on the same side as *p*_*b*_ among two adjacent wedges of a line segment (*p*_*a*_, *p*_*F*_). Accordingly, we denote *W*_2_, …, *W*_6_ in clockwise order.

**Lemma 8**. *If* $I_{a}\left(C_{b}^{j},C_{c}^{k}\right)\in \left(W_{1}\cup W_{2}\right)$ *and* $I_{a}\left(C_{b}^{j},C_{c}^{k}\right)\notin C_{a}^{i}$, *no convex cell* $CV(C_{a}^{i-2\delta_{N}},C_{b}^{j+\delta_{N}},C_{c}^{k+\delta_{N}})$ *exists where δ*_*N*_ < *i*/2, *and* $i,j,k,\delta_{N}\in N$.

*Proof*. Given a convex cell, we consider two cases: the first case is when one of vertices of the convex cell is closer to *p*_*a*_ $\left(I_{a}\left(C_{b}^{j},C_{c}^{k}\right)\in \Delta p_{a}p_{b}p_{c}\right)$ and the second case is when the closest point to *p*_*a*_ is on the boundary. In the first case, we use $I_{a}\left(C_{b}^{j},C_{c}^{k}\right)$ to determine whether $C_{a}^{i}$ contains $I_{a}\left(C_{b}^{j},C_{c}^{k}\right)$. In the case that $I_{a}\left(C_{b}^{j},C_{c}^{k}\right)\notin \Delta p_{a}p_{b}p_{c}$, the intersection point of $CV(C_{a}^{i},C_{b}^{j},C_{c}^{k})$ and the boundary of Δ*p*_*a*_*p*_*b*_*p*_*c*_ is used for proof instead of $I_{a}\left(C_{b}^{j},C_{c}^{k}\right)$.

**Fig 6:**
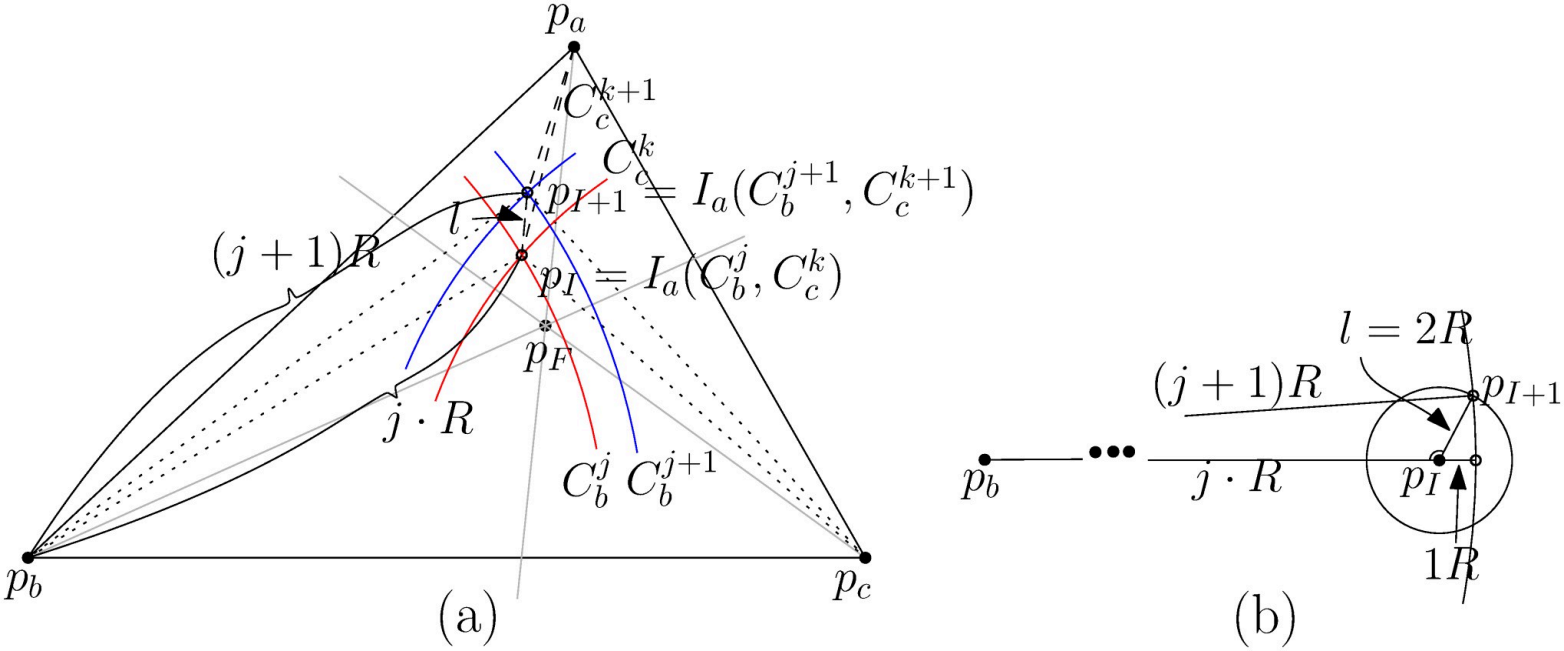
(a) The distance *l* between the intersection point $p_{I}=I_{a}\left(C_{b}^{j},C_{c}^{k}\right)$ of $C_{b}^{j}$ and $C_{c}^{k}$, and $p_{I+1}=I_{a}\left(C_{b}^{j+1},C_{c}^{k+1}\right)$. (b) For *l* ≥ 2*R*, the angle ${∠}_{p_{b}p_{I}p_{I+1}}$ is strictly less than $\frac{2}{3}\pi$.

We explain with the case that $I_{a}\left(C_{b}^{j},C_{c}^{k}\right)\in \Delta p_{a}p_{b}p_{c}$. We show that |*p*_*a*_*p*_*I*_| − 2*R* ≤ |*p*_*a*_*p*_*I*+1_| for any $p_{I}=I_{a}\left(C_{b}^{j},C_{c}^{k}\right),j\geq j_{F},k\geq k_{F}$, and $p_{I+1}=I_{a}\left(C_{b}^{j+1},C_{c}^{k+1}\right)$ which implies that *p*_*I*+1_ cannot be contained by $C_{a}^{i-2}$ unless *p*_*I*_ lies inside $C_{a}^{i}$. As depiced in Fig 6, we denote *l* = |*p*_*I*_*p*_*I*+1_| as the distance between *p*_*I*_ and *p*_*I*+1_. If *l* ≥ 2*R*, $∠p_{b}p_{I}p_{I+1}<\frac{2}{3}\pi$ (see Fig 6(b)). Since *p*_*I*_ ∈ *W*_1_ ∪ *W*_2_, $∠p_{b}p_{I}p_{c}<\frac{2}{3}$. Thus, it should hold that $∠p_{b}p_{I}p_{I+1}+∠p_{c}p_{I}p_{I+1}>\frac{4}{3}\pi$. It implies that *l* < 2*R*. *l* + |*p*_*a*_*p*_*I*+1_| ≥ |*p*_*a*_*p*_*I*_| by triangle inequality. It holds that |*p*_*a*_*p*_*I*+1_| ≥ |*p*_*a*_*p*_*I*_| − *l* ≥ |*p*_*a*_*p*_*I*_| − 2*R*.

Consequently, $CV(C_{a}^{i-2\delta_{N}},C_{b}^{j+\delta_{N}},C_{c}^{k+\delta_{N}})$ does not exist.

Let *EP*_*b*,*c*_(*p*_*x*_) be the ellipse through a point *p*_*x*_ with two focal points *p*_*b*_ and *p*_*c*_ (see Fig 5). Let *HB*_*b*,*c*_(*p*_*x*_) be the *p*_*c*_-side curve of the hyperbola with two focal points *p*_*b*_ and *p*_*c*_ through a point *p*_*x*_.

**Lemma 9**. *If* $I_{a}\left(C_{b}^{j},C_{c}^{k}\right)\in W_{1}$ (or *W*_2_) *and* $I_{a}\left(C_{b}^{j},C_{c}^{k}\right)\notin C_{a}^{i}$, *no convex cell* $CV(C_{a}^{i-\delta_{N}},C_{b}^{j},C_{c}^{k+\delta_{N}})$ (*or* $CV(C_{a}^{i-\delta_{N}},C_{b}^{j+\delta_{N}},C_{c}^{k})$), $\delta_{N}<i,\delta_{N}\in N$, *exists*.

**Fig 7:**
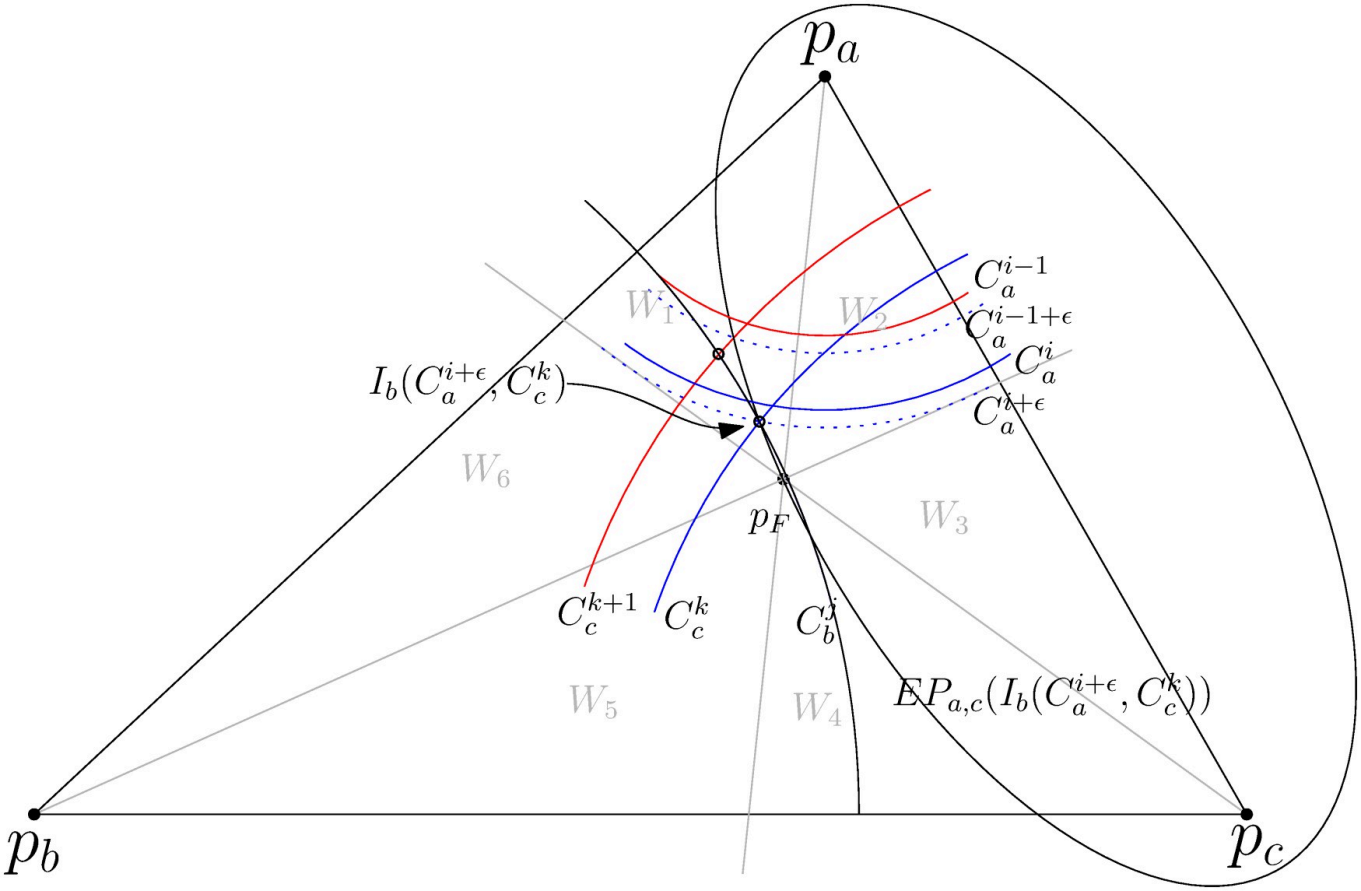
When one of intersection points between *EP*_*b*,*c*_(*p*_*x*_) and $C_{b}^{j}$ where *j* ⋅ *R* > |*p*_*b*_*p*_*F*_| lies inside of *W*_1_, the other point is inside of *W*_2_ ∪ *W*_3_ ∪ *W*_4_ if it exists inside of the triangle.

*Proof*. We consider a circle $C_{a}^{i+\epsilon }$ centered at *p*_*a*_ with a radius of $\left(i+\epsilon \right)R=|p_{a}I_{a}\left(C_{b}^{j},C_{c}^{k}\right)|$ and an ellipse $EP_{a,c}\left(I_{b}\left(C_{a}^{i+\epsilon },C_{b}^{j}\right)\right)$ as illustrated in Fig 7. Note that $I_{a}\left(C_{b}^{j},C_{c}^{k}\right)=I_{b}\left(C_{a}^{i+\epsilon },C_{c}^{k}\right)$. When one of the intersection points of the ellipse and the circle lies inside of *W*_1_, the other intersection point is located in *W*_2_ ∪ *W*_3_ ∪ *W*_4_ since |*p*_*b*_*p*_*F*_| ≤ *j* ⋅ *R*. It implies that $I_{a}\left(C_{b}^{j},C_{c}^{k+\delta }\right)\in \Delta p_{a}p_{b}p_{c},\delta >0$ is outside of $EP_{a,c}\left(I_{b}\left(C_{a}^{i+\epsilon },C_{c}^{k}\right)\right)$ as well as $EP_{a,c}\left(I_{b}\left(C_{a}^{i-\delta },C_{c}^{k+\delta }\right)\right)$. Thus, $CV(C_{a}^{i-\delta_{N}},C_{b}^{j},C_{c}^{k+\delta_{N}})$ does not exist unless $I_{a}\left(C_{b}^{j},C_{c}^{k}\right)\in C_{a}^{i}$.

Now, we look into the cells in regions 2, 3, and 4. W.l.o.g., we show that there is no better convex cell compared to the candidate cell of the algorithm in region 2. We assume that |*p*_*b*_*p*_*F*_| ≥ |*p*_*c*_*p*_*F*_| for the proof of Lemmas 10 and 11.

**Lemma 10**. *No convex cell* $CV(C_{a}^{i},C_{b}^{j},C_{c}^{k})$ *exists such that i* + *j* + *k* < i* + j* + k*, *i* ≤ *i*_*F*_, *j* ≥ *j*_*F*_, *and k* ≥ *k*_*F*_ *where* $CV(C_{a}^{i^{*}},C_{b}^{j^{*}},C_{c}^{k^{*}})$ *is the candidate cell as the result of* AlgB.

*Proof*. *AlgB* investigates $CV(C_{a}^{i},C_{b}^{j_{F}},C_{c}^{k_{F}+2})$, $CV(C_{a}^{i},C_{b}^{j_{F}},C_{c}^{k_{F}+1})$, $CV(C_{a}^{i},C_{b}^{j_{F}},C_{c}^{k_{F}})$, and $CV(C_{a}^{i},C_{b}^{j_{F}+1},C_{c}^{k_{F}})$ by solving Inequality (7) in Step 2-1. We prove that there is no better cell in region 2 by comparing it with these four convex cells.

*EP*_*b*,*c*_(*p*_*F*_) is tangent to the circle centered at *p*_*a*_ with a radius of |*p*_*a*_*p*_*F*_| and tangent lines of *EP*_*b*,*c*_(*p*_*F*_) and *HB*_*b*,*c*_(*p*_*F*_) are orthogonal at a point *p*_*F*_. Thus, $\bar{p_{a}p_{F}}$ becomes a tangent line of *HB*_*b*,*c*_(*p*_*F*_) at *p*_*F*_ and *HB*_*b*,*c*_(*p*_*F*_) lies in *W*_2_ ⋃ *W*_3_ ⋃ *W*_4_. Since |*p*_*b*_*p*_*F*_| − |*p*_*c*_*p*_*F*_| < (*j*_*F*_ + 1 − *k*_*F*_)*R*, $HB_{b,c}\left(I_{a}\left(C_{b}^{j_{F}+1},C_{c}^{k_{F}}\right)\right)$ is also in *W*_2_ ⋃ *W*_3_ ⋃ *W*_4_. Thus, $I_{a}\left(C_{b}^{j_{F}+1},C_{c}^{k_{F}}\right)$ is in *W*_2_. By Lemma 9, convex cells composed by $C_{c}^{k_{F}}$ and $C_{b}^{j_{F}+1+\delta_{N}}$, $\delta_{N}\in N$ require at least the same number of Steiner points as that of the convex cell consisting of $C_{b}^{j_{F}+1}$ and $C_{c}^{k_{F}}$.

$I_{a}\left(C_{b}^{j_{F}},C_{c}^{k_{F}+2}\right)$ is in *W*_1_ because *j*_*F*_*R* − |*p*_*b*_*p*_*F*_| < *R* and the distance between two intersection points of *W*_2_ and $C_{b}^{j_{F}}$ is less than $\sqrt{3}R$. By Lemma 9, convex cells composed by $C_{b}^{j_{F}}$ and $C_{c}^{k_{F}+2+\delta_{N}},\delta_{N}\in N$ require Steiner points not less than that of the convex cell consisting of $C_{b}^{j_{F}}$ and $C_{c}^{k_{F}+2}$.

Up to now, we have examined the convex cells $CV(C_{a}^{i},C_{b}^{j},C_{c}^{k})$ such that *i* ≤ *i*_*F*_ and either *j* = *j*_*F*_ or *k* = *k*_*F*_. Next, we consider a convex cell $CV(C_{a}^{i},C_{b}^{j},C_{c}^{k})$ such that *i* ≤ *i*_*F*_, *j* = *j*_*F*_ + *δ*1_*N*_, *k* = *k*_*F*_ + *δ*2_*N*_, and $\delta 1_{N},\delta 2_{N}\in N$. When *δ*1_*N*_ ≥ *δ*2_*N*_, we compare the convex cell $CV(C_{a}^{i},C_{b}^{j},C_{c}^{k})$ with the convex cell *CV*_*comp*_ composed by $C_{b}^{j_{F}+\delta 1_{N}-\delta 2_{N}}$ and $C_{c}^{k_{F}}$. By Lemma 8, $CV(C_{a}^{i},C_{b}^{j},C_{c}^{k})$ is not better than *CV*_*comp*_ which is already checked above. Equally, we compare the convex cell $CV(C_{a}^{i},C_{b}^{j},C_{c}^{k})$ with the convex cell *CV*_*comp*_ composed by $C_{b}^{j_{F}}$ and $C_{c}^{k_{F}+\delta 2_{N}-\delta 1_{N}}$ when *δ*1_*N*_ < *δ*2_*N*_. By Lemma 8, $CV(C_{a}^{i},C_{b}^{j},C_{c}^{k})$ is also not better than *CV*_*comp*_. Consequently, there does not exist $CV(C_{a}^{i},C_{b}^{j},C_{c}^{k})$ such that *i* + *j* + *k* < i* + j* + k*, *i* ≤ *i*_*F*_, *j* ≥ *j*_*F*_, and *k* ≥ *k*_*F*_ where $CV(C_{a}^{i^{*}},C_{b}^{j^{*}},C_{c}^{k^{*}})$ is the candidate cell as the result of *AlgB*.

Lastly, we prove that there is no better convex cell in regions 5, 6, and 7 than the candidate cell. W.l.o.g., we show the case of region 5.

**Lemma 11**. *No convex cell* $CV(C_{a}^{i},C_{b}^{j},C_{c}^{k})$ exists such that *i* + *j* + *k* < i* + j* + k*, *i* > *i*_*F*_, *j* < *j*_*F*_, and *k* < *k*_*F*_ where $CV(C_{a}^{i^{*}},C_{b}^{j^{*}},C_{c}^{k^{*}})$ is the candidate cell as the result of *AlgB*.

*Proof*. *HB*_*b*,*c*_(*p*_*F*_) is tangent to $\bar{p_{a}p_{F}}$ at *p*_*F*_ as explained in Lemma 10. Since the algorithm investigates all convex cells of which one of vertices is on $HB_{b,c}\left(I_{a}\left(C_{b}^{j},C_{c}^{k}\right)\right),j_{F}-k_{F}-2\leq j-k\leq j_{F}-k_{F}+1$, we first look up convex cells $CV(C_{a}^{i},C_{b}^{j},C_{c}^{k})$ such that *i* > *i*_*F*_, *j* < *j*_*F*_, *k* < *k*_*F*_, and *j* − *k* < *j*_*F*_ − *k*_*F*_ − 2 and then $CV(C_{a}^{i},C_{b}^{j},C_{c}^{k})$ such that *i* > *i*_*F*_, *j* < *j*_*F*_, *k* < *k*_*F*_, and *j* − *k* > *j*_*F*_ − *k*_*F*_ + 1. If an ellipse with two focal points *p*_*b*_ and *p*_*c*_ intersects with $C_{a}^{i},i>i_{F}$ at a single point, the point is in the same side of *HB*_*b*,*c*_(*p*_*F*_) as *p*_*c*_. When the ellipse intersects with $C_{a}^{i},i>i_{F}$ at two points, if one intersection point is on the same side of *HB*_*b*,*c*_(*p*_*F*_) as *p*_*b*_, the other intersection point should be in the opposite side of *HB*_*b*,*c*_(*p*_*F*_). Therefore, if there exists a convex cell $CV(C_{a}^{i},C_{b}^{j},C_{c}^{k})$ such that *i* + *j* + *k* < i* + j* + k*, *i* > *i*_*F*_, *j* < *j*_*F*_, *k* < *k*_*F*_, and *j* − *k* < *j*_*F*_ − *k*_*F*_ − 2, there also exists a convex cell $CV(C_{a}^{i},C_{b}^{j^{\prime }},C_{c}^{k^{\prime }})$ such that *j*′ + *k*′ is equal to *j* + *k* and *j*′ − *k*′ is either *j*_*F*_ − *k*_*F*_ − 1 or *j*_*F*_ − *k*_*F*_ − 2. Since the algorithm investigates $HB_{b,c}\left(I_{a}\left(C_{b}^{j_{F}-1},C_{c}^{k_{F}}\right)\right)$ and $HB_{b,c}\left(I_{a}\left(C_{b}^{j_{F}-2},C_{c}^{k_{F}}\right)\right)$, $CV(C_{a}^{i},C_{b}^{j},C_{c}^{k})$ cannot be better than the candidate cell of the algorithm.

Next, we inspect convex cells $CV(C_{a}^{i},C_{b}^{j},C_{c}^{k})$ such that *i* > *i*_*F*_, *j* < *j*_*F*_, *k* < *k*_*F*_, and *j* − *k* > *j*_*F*_ − *k*_*F*_ + 1. Since all intersection points of $HB_{b,c}\left(I_{a}\left(C_{b}^{j_{F}+1},C_{c}^{k_{F}}\right)\right)$ and $C_{b}^{j},0<j<j_{F}$ are in *W*_4_, any convex cell $CV(C_{a}^{i},C_{b}^{j},C_{c}^{k})$ such that *i* > *i*_*F*_, *j* < *j*_*F*_, *k* < *k*_*F*_, and *j* − *k* > *j*_*F*_ − *k*_*F*_ + 1 cannot be better than $CV(C_{a}^{i},C_{b}^{j},C_{c}^{k})$ induced by $HB_{b,c}\left(I_{a}\left(C_{b}^{j_{F}+1},C_{c}^{k_{F}}\right)\right)$ by Lemma 9 with a slight modification.

**Theorem 2**. *Given 3 input points*, AlgB *computes an optimal Steiner tree for STP-MSPBEL in constant time*.

*Proof*. It is obvious that *AlgB* takes constant time. We can compute the Fermat point in constant time in Step 1. In Step 2, we solve Eq (6) at most 8 times for each sub-part. Finally, in Step 3, the minimum spanning tree of three points can also be constructed in constant time.

Now, we show that the result steinerized tree is optimal for STP-MSPBEL. There are two ways to connect three terminal points with minimum Steiner points: one is to connect three points with a junction point, and the other is to connect two pairs of input points directly. Through Step 2, the algorithm presents the optimal 3-star tree among all possible 3-star trees connecting input points. By Observations 1 and 3, at least one optimal convex cell for a junction point intersects with Δ*p*_*a*_*p*_*b*_*p*_*c*_. By Lemmas 9, 10, and 11, we have shown that the result convex cell of the algorithm is better than or equal to other convex cells in Δ*p*_*a*_*p*_*b*_*p*_*c*_. Therefore, an optimal 3-star Steiner tree can be constructed by putting the junction point in the result convex cell and connecting input points with the minimum number of Steiner points. Since the algorithm computes the minimum spanning tree in Step 3, the optimal Steiner tree for STP-MSPBEL is presented by comparing those two trees.

**Fig 8:**
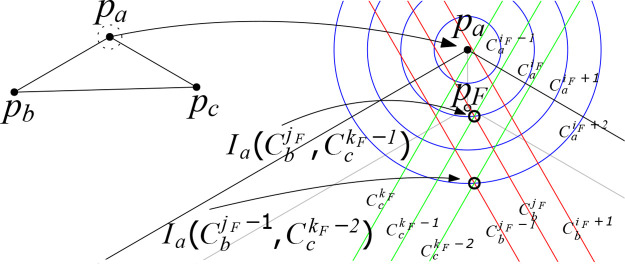
The location of a junction point for an optimal 3-star tree can be found outside of $C_{a}^{i_{F}+1}$ when *R* = 1, |*p*_*a*_*p*_*F*_| = 1.739, |*p*_*b*_*p*_*F*_| = 1100.95 and |*p*_*c*_*p*_*F*_| = 1000.3007. It shows that none of the intersection points between $C_{a}^{i_{F}}$, $C_{a}^{i_{F}-1}$, $C_{b}^{j_{F}}$, $C_{b}^{j_{F}-1}$, $C_{c}^{k_{F}}$, and $C_{c}^{k_{F}-1}$ might yield the optimal junction point location.

We argue that the optimal location for the junction point may not be found although investigating all intersection points among $C_{a}^{i_{F}}$, $C_{a}^{i_{F}-1}$, $C_{b}^{j_{F}}$, $C_{b}^{j_{F}-1}$, $C_{c}^{k_{F}}$, and $C_{c}^{k_{F}-1}$ which implies that the method in [18] cannot provide the optimal Steiner tree for three points. The counterexample exists when |*p*_*a*_*p*_*F*_| is short, and both |*p*_*b*_*p*_*F*_| and |*p*_*c*_*p*_*F*_| are relatively long. As shown in Fig 8, we set *R* = 1, |*p*_*a*_*p*_*F*_| = 1.739, |*p*_*b*_*p*_*F*_| = 1100.95 and |*p*_*c*_*p*_*F*_| = 1000.3007. Then, the distance between *p*_*a*_ and $I_{a}\left(C_{b}^{j_{F}},C_{c}^{k_{F}-1}\right)$ is greater than 2; however,the distance between *p*_*a*_ and $I_{a}\left(C_{b}^{j_{F}-1},C_{c}^{k_{F}-2}\right)$ is less than 4. Thus, if the junction point is located at $I_{a}\left(C_{b}^{j_{F}-1},C_{c}^{k_{F}-2}\right)$, it requires 2101 Stiner points which is one less than that using $I_{a}\left(C_{b}^{j_{F}},C_{c}^{k_{F}-1}\right)$. If the junction point is located at any intersection points among $C_{a}^{i_{F}}$, $C_{a}^{i_{F}-1}$, $C_{b}^{j_{F}}$, $C_{b}^{j_{F}-1}$, $C_{c}^{k_{F}}$, and $C_{c}^{k_{F}-1}$, it requires at least 2102 Steiner points. In addition, the steinerized minimum spanning tree needs 2102 Steiner points.

## 5 A combined algorithm

**Fig 9:**
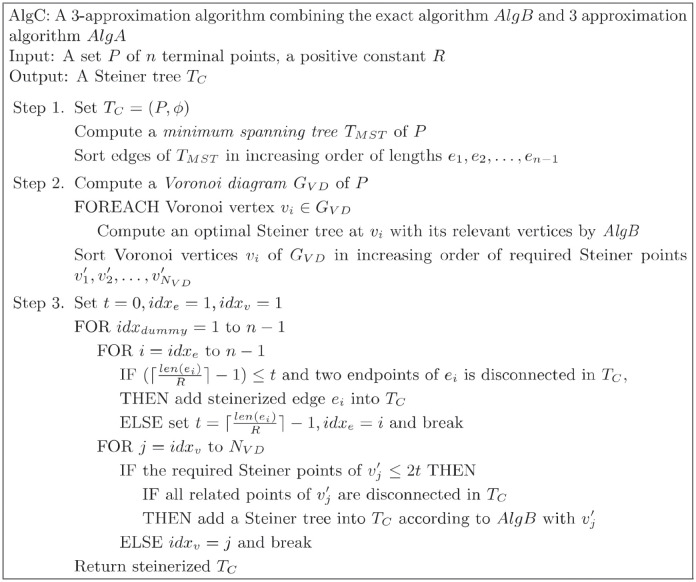
*AlgC*: A combined algorithm.

In this section, we develop another 3-approximation algorithm by combining the exact algorithm *AlgB* and the approximation algorithm *AlgA* to further reduce the number of required Steiner points in *O*(*n* log *n*) time. As described in Fig 9, *AlgC* computes the minimum spanning tree (MST) of terminal points and sorts MST edges by the number of required Steiner points in ascending order. Next, *AlgC* constructs the Voronoi diagram of the set of terminal points as sites and calculates the number of Steiner points required at each Voronoi vertex to connect its relevant 3 terminal points by *AlgB*. Then, the algorithm sorts the Voronoi vertices by the number of required Steiner points. Let *E*_*MST*_(*i*) be a set of MST edges requiring *i* Steiner points and *V*_*VD*_(*j*) be a set of Voronoi vertices requiring *j* Steiner points. By looking up the sorted lists of MST edges and Voronoi vertices, we are able to construct a steinerized spanning tree. We process an edge case or a vertex case by the following rules:

Rule 1. *E*_*MST*_(*i*) is processed before *V*_*VD*_(*j*) where *i* < ⌈*j*/2⌉.
Rule 2. *V*_*VD*_(*j*) is processed before *E*_*MST*_(*i*) where *j* ≤ 2*i*.

In the edge case, if two endpoints are not in the same component, we steinerize the edge and add it to the resulting tree. In the vertex case, if three relevant terminal points of the Voronoi vertex are not in the same component, we add Steiner points and relevant edges into the resulting tree by *AlgB*. For example, at first, we add all MST edges in *E*_*MST*_(0) by Rule 1, and then we look at the Voronoi vertices in *V*_*VD*_(1) and *V*_*VD*_(2) by Rule 2. Iteratively, we increase the result tree size by processing with *E*_*MST*_(*i*), *V*_*VD*_(2*i* + 1), and *V*_*VD*_(2*i* + 2). The basic idea behind this strategy is that if three points are in different connected components after adding edges in *E*_*MST*_(*i*), at least 2(*i* + 1) Steiner points are required to connect those three components by MST edges. Thus, it is beneficial to use the vertex case if *V*_*VD*_(2*i* + 1) or *V*_*VD*_(2*i* + 2) connects those three points. If three points are already in the same connected component before *V*_*VD*_(*j*) are considered, the bottleneck of the path between any of two points is less than or equal to ⌈*j*/2⌉ − 1. Thus, Despite removing the two bottleneck edges, the result tree deteriorates when Steiner points are inserted based on *V*_*VD*_(*j*).

**Theorem 3**. AlgC *provides the Steiner tree T*_*C*_ *satisfying C*(*T*_*C*_) ≤ *C*(*T*_*A*_) *in O*(*n* log *n*) *time where T*_*A*_ *is the Steiner tree produced by* AlgA.

*Proof*. It is straightforward that *AlgC* takes *O*(*n* log *n*) time. Since *AlgB* takes constant time, Step 1 and 2 of *AlgC* takes *O*(*n* log *n*) time. In Step 3, there are *n* − 1 MST edges and the *O*(*n*) Voronoi vertices in the sorted lists. Since each connectivity query takes *O*(log *n*) time, the overall time complexity is bounded by *O*(*n* log *n*). The performance of *AlgC* is better than or equal to that of *AlgA*. *AlgC* first processes with *E*(0) and *V*(1). The resulting tree (forest) is exactly the same as the result of *AlgA* after processing Step 3. In the context of *AlgC*, Steiner points are introduced through the vertex case exclusively if the spanning tree established by *AlgB* outperforms the tree formed by steinerized edges of the minimum spanning tree. Consequently, *AlgC* produces a spanning tree that requires an equal or fewer number of Steiner points than that of *AlgA*.

## 6 Conclusion

We present approximation algorithms for the Steiner tree problem with the minimum number of Steiner points and bounded edge length. Previously, the best-known deterministic approximation algorithm has *O*(*n*^3^) running time with an approximation ratio of 3. In this paper, we propose *O*(*n* log *n*)-time approximation algorithm with the same approximation ratio, significantly improving the time complexity. Additionally, to enhance performance, we introduce the first exact algorithm that can provide an optimal Steiner tree for any given three points in constant time. By using this exact algorithm, we develop another 3-approximation algorithm that runs in *O*(*n* log *n*) time with better performance in terms of the number of required Steiner points.

## References

[1] Lin GH, Xue G. Steiner tree problem with minimum number of Steiner points and bounded edge-length. Information Processing Letters. 1999;69(2):53–57. doi: 10.1016/S0020-0190(98)00201-4

[2] Gilbert EN, Pollak HO. Steiner Minimal Trees. SIAM Journal on Applied Mathematics. 1968;16(1):1–29. doi: 10.1137/0116001

[3] Cheng X, Du DZ, Wang L, Xu B. Relay sensor placement in wireless sensor networks. Wireless Networks. 2008;14(3):347–355. doi: 10.1007/s11276-006-0724-8

[4] Younis M, Senturk IF, Akkaya K, Lee S, Senel F. Topology management techniques for tolerating node failures in wireless sensor networks: A survey. Computer Networks. 2013;.

[5] Hartmanis J. Computers and intractability: a guide to the theory of np-completeness (michael r. garey and david s. johnson). Siam Review. 1982;24(1):90. doi: 10.1137/1024022

[6] Chlebík M, Chlebíková J. The Steiner tree problem on graphs: Inapproximability results. Theoretical Computer Science. 2008;406(3):207–214. doi: 10.1016/j.tcs.2008.06.046

[7] Byrka J, Grandoni F, Rothvoß T, Sanità L. Steiner tree approximation via iterative randomized rounding. Journal of the ACM (JACM). 2013;60(1):1–33. doi: 10.1145/2432622.2432628

[8] Chen CY. An efficient approximation algorithm for the Steiner tree problem. In: Proceedings of the 2nd International Conference on Information Science and Systems; 2019. p. 179–184.

[9] Chen CY, Hsieh SY. An improved algorithm for the Steiner tree problem with bounded edge-length. Journal of Computer and System Sciences. 2022;123:20–36. doi: 10.1016/j.jcss.2021.07.003

[10] Traub V, Zenklusen R. Local search for weighted tree augmentation and Steiner tree. In: Proceedings of the 2022 Annual ACM-SIAM Symposium on Discrete Algorithms (SODA). SIAM; 2022. p. 3253–3272.

[11] Zhang J, Liu Z, Deng X, Yin J. Truthful Mechanisms for Steiner Tree Problems. In: Proceedings of the AAAI Conference on Artificial Intelligence. vol. 37; 2023. p. 5884–5891.

[12] Berman P, Karpinski M, Zelikovsky A. 25-Approximation Algorithm for Steiner Tree Problem with Distances 1 and 2. In: Workshop on Algorithms and Data Structures. Springer; 2009. p. 86–97.

[13] Chen D, Du DZ, Hu XD, Lin GH, Wang L, Xue G. Approximations for Steiner trees with minimum number of Steiner points. Journal of Global Optimization. 2000;18(1):17–33. doi: 10.1023/A:1008384012064

[14] Senel F, Younis M. Relay node placement in structurally damaged wireless sensor networks via triangular steiner tree approximation. Computer Communications. 2011;34(16):1932–1941.

[15] Senel F, Younis M. Optimized relay node placement for establishing connectivity in sensor networks. In: Global Communications Conference (GLOBECOM), 2012 IEEE; 2012. p. 512–517.

[16] Shin D. Efficient algorithms with performance guarantees for geometric problems in wireless networks; 2016. Doctoral dissertation, KAIST, http://library.kaist.ac.kr/search/detail/view.do?bibCtrlNo=648280.

[17] Gueron S, Tessler R. The fermat-steiner problem. The American Mathematical Monthly. 2002;109(5):443–451. doi: 10.1080/00029890.2002.11919871

[18] Senel F, Younis M. Novel relay node placement algorithms for establishing connected topologies. Journal of Network and Computer Applications. 2016;70:114–130. doi: 10.1016/j.jnca.2016.04.025

[19] Mark dB, Otfried C, Marc vK, Mark O. Computational geometry algorithms and applications. Spinger; 2008.

[20] Sack JR, Urrutia J. Handbook of computational geometry. Access Online via Elsevier; 1999.

[21] Hwang FK, Richards DS, Winter P. The Steiner tree problem. Elsevier; 1992.

[22] Leiserson CE, Rivest RL, Stein C, Cormen TH. Introduction to algorithms. The MIT press; 2001.

